# Genetic and pharmacological targeting of nicotinic acetylcholine receptor action blocks tumor progression in mouse models of breast cancer

**DOI:** 10.1093/jimmun/vkaf148

**Published:** 2025-07-14

**Authors:** Matthew A Heard, Jin Qian, Sakeef Sayeed, Sereena Mechlowicz, Qingyang Zhang, Sudha Yeluri, Katie Pool, Ryan Yamane, Gerald P Morris, Brian P Eliceiri

**Affiliations:** Department of Surgery, University of California San Diego, La Jolla, CA, United States; Department of Surgery, University of California San Diego, La Jolla, CA, United States; Department of Surgery, University of California San Diego, La Jolla, CA, United States; Department of Surgery, University of California San Diego, La Jolla, CA, United States; Department of Pathology, University of California San Diego, La Jolla, CA, United States; Department of Surgery, University of California San Diego, La Jolla, CA, United States; Department of Dermatology, University of California San Diego, La Jolla, CA, United States; Department of Surgery, University of California San Diego, La Jolla, CA, United States; Department of Pathology, University of California San Diego, La Jolla, CA, United States; Department of Surgery, University of California San Diego, La Jolla, CA, United States; Department of Dermatology, University of California San Diego, La Jolla, CA, United States

**Keywords:** nicotinic acetycholine receptor, dendritic cells, triple negative breast cancer, immunotherapeutics, metastasis

## Abstract

Effective small molecule therapies are a major unmet need in triple-negative breast cancer. Therefore, we examined the mechanism of action of a novel cancer therapeutic target in preclinical mouse models focusing on the α7 nicotinic acetylcholine receptor (CHRNA7). E0771 breast tumor cells were implanted into CHRNA7^KO^ mice to determine the role of CHRNA7, which is expressed in tumor-associated myeloid immune cells. We observed that tumor-bearing CHRNA7^KO^ mice had decreased survival and increased tumor burden linked to a CHRNA7-mediated reduction in immune cell activation. Based on the tumor permissive phenotype of CHRNA7^KO^ mice, we tested the effect of a small molecule agonist of CHRNA7, AR-R17779, in several mouse models of breast cancer. For example, in both the E0771 tumor model and PyMT tumor models, treatment with AR-R17779 increased survival. In the 4T1 breast tumor model, treatment with AR-R17779 also increased survival, with a well-defined reduction in primary tumor burden and lung metastases. The antitumorigenic effects of AR-R17779 were linked to an adaptive immune response based on in vivo studies showing a survival benefit when AR-R17779 was administered as a combination therapy with anti-PD-L1, demonstrating that the effects of AR-R17779 were dependent on CD8 T cells, and in vitro studies showing AR-R17779 treatment of dendritic cells increased T cell activation. Together these findings supported the importance of CHRNA7 as a novel therapeutic target expressed on dendritic cells based on its role in potentiating the adaptive immune response in mouse models of breast cancer.

## Introduction

The evasion of immunosurveillance is a hallmark of cancer, characterized by compromised immune cell signaling.^1^ Current immunotherapeutics harness the adaptive immune system to reduce tumor burden in many cancers, but this has not yet been demonstrated for breast cancer.^2^ While immune cells such as lymphocytes depend on priming and antigen presentation, there are few examples of targeting pathways that enhance the function of antitumorigenic myeloid immune cells such as monocytes, macrophages, or dendritic cells (DCs). Given the unmet need for novel therapeutics, low survival, limited treatment options, and poor understanding of mechanisms regulating immune cell activation, we focus on triple-negative breast cancer (TNBC)^3^^,^^4^ and demonstrate the ability to harness the adaptive immune response in breast cancer. In this study, we demonstrate a reduction in TNBC tumor burden by targeting a novel pathway involving α7 nicotinic acetylcholine receptor (CHRNA7) using AR-R17779, a small molecule agonist that is highly specific for CHRNA7.^5–7^

The functional role for CHRNA7 in monocytes, macrophages, and to a lesser degree, antigen-presenting cells (APCs) has been established in many studies in models of injury and infection.^8^^,^^9^ Although there are many studies on nicotine, tobacco-related carcinogens, and nicotinic receptors in tumor cells outside the focus of our study, no studies to date have identified a role for CHRNA7 in tumor-associated host immune cells.^10^ Additionally, there have been multiple studies linking sympathetic and parasympathetic nervous system signaling to tumor growth and metastasis, prompting further interest on the effect of AR-R17779 to modulate the tumor microenvironment.^11^ Our focus is on the action of CHRNA7 in the host compartment, specifically immune cells, with a potential for high impact based on the availability of a small molecule drug agonist of CHRNA7. Previous studies have shown that CHRNA7 is a mediator of myeloid immune cell signaling.^8^^,^^9^ For example, studies outside the field of cancer have established the functional relevance of CHRNA7 in tissue macrophages, but this has not been established in tumor-associated immune cells. Based on CHRNA7 expression in monocytes, macrophages, and DCs, we focused on immune competent mouse models of TNBC to determine the functional role of CHRNA7 in mediating immune cell activation in tumor progression. We provide evidence here that CHRNA7 functions as a novel therapeutic target in tumor-associated immune cells, with a particular focus on tumor-associated APCs. A combination of genetic and pharmacological approaches is used to test the hypothesis that CHRNA7 has a functional role in driving the activation of APCs to promote antitumor activity of lymphocytes. Previous pharmacological testing, along with lack of effectiveness of existing immune checkpoint therapies in TNBC, have driven us to compare monotherapy targeting CHRNA7 versus combination therapy with an immune checkpoint inhibitor. Our results are consistent with the hypothesis that CHRNA7 action is dependent on APCs through an alternate pathway, ultimately reducing tumor growth and increasing survival. Here, we show that addition of an immune checkpoint inhibitor substantially increases the antitumor activity of a CHRNA7 agonist in mouse models of TNBC.

## Materials and methods

### Animal tumor studies

All studies were approved by the University of California San Diego Animal Care and Use Committee (S07339). For studies of E0771 tumor growth, tumor cells were implanted into 8- to 12-wk-old CHRNA7^KO^ mice (B6.129S7-*Chrna7^tm1Bay^/*J) versus sibling-matched wild-type (WT) littermates that were generated by Dr. A. Beaudet^12^ and purchased through the Jackson Laboratory (RRID: IMSR_JAX:003232). Injections of the fourth mammary fat pad (MFP) were performed with 1 × 10^6^ E0771 mammary adenocarcinoma cells (ATCC; #CRL-3461, RRID: CVCL_GR23) cultured in RPMI 1640 in 10% fetal calf serum as previously described.^13^ Similarly, for the AR-R17779 studies of E0771 tumor progression, 1 × 10^6^ E0771 cells were injected into 8- to 12 wk-old mice except that C57BL/6 female mice were used (RRID: IMSR_JAX:000664). For survival studies testing monotherapy and combination therapy in the E0771 model, 7.5 × 10^5^ E0771 cells were implanted into the MFP to reduce the risk of ulceration over a longer time point. We observed that tumor onset following injection of E0771 tumor cells was substantially faster in C57BL/6 mice (RRID: IMSR_JAX:000664) than in the WT siblings of the CHRNA7^KO^ mice (RRID: IMSR_JAX:003232), possibly due to effects of the mixed B6/129 background of the founders of the CHRNA7 line that remained after multiple rounds (n > 5) of backcrossing. For tumor studies using 4T1 tumor cells (ATCC; #CRL-2539, RRID: CVCL_0125) 8- to 12-wk-old female BALB/c mice (RRID: IMSR_JAX000651) were subjected to MFP injections with 1 × 10^5^ 4T1 tumor cells. For lung metastasis assays in the 4T1 model, primary MFP tumors were first resected when tumors were palpable (10–14 d) and randomized for treatment with AR-R17779 or saline as a vehicle control. Following recovery, mice were housed for an additional 10 d, euthanized, and the lungs harvested into Bouin’s fixative (Sigma-Aldrich; #HT10132). Lung macrometastases were then enumerated by a double-blinded observer with a stereoscope. For spontaneous breast tumor studies, MMTV-PyMT female mice (RRID: IMSR_JAX002374) were bred and randomized for treatment with AR-R17779 versus saline control when multifocal breast tumors were observed. Treatment with AR-R17779 (Sigma-Aldrich; #SML2049) for all tumor studies was performed by intraperitoneal injection (1 mg/kg) administered every 2 d using saline as a vehicle control. Mouse survival and tumor burden were assessed every 3 to 5 d and the kinetics of tumor growth determined with calipers where tumor volume = 1/2 (length × width^2^).^14^ Wet tumor weights were assessed upon euthanasia, with tumors analyzed by immunohistochemistry, flow cytometry, and cell separation as described subsequently. For in vitro assays of CHRNA7 expression and bone marrow–derived dendritic cell (BMDC) studies using the CHRNA7^KO^ line both male and female mice were used as cell donors.

### Immunohistochemistry

Tumor tissues resected upon harvest were embedded in formalin, 10 µm sections, subjected to antigen retrieval with Signal Stain Citrate Unmasking solution (Cell Signaling Technology; #14746), incubated with anti-CD11c antibody (Cell Signaling Technology; #97585S), and detected with a horseradish peroxidase detection system, Signal Stain Boost, per the manufacturer’s protocol (Cell Signaling Technology; #8112). Slides were imaged with an FSX100 light microscope (Olympus America).

### Flow cytometry

Tumors and spleens were processed by enzymatic digestion to obtain single cells using the Tumor Dissociation Kit (Miltenyi Biotec; #130-096-730) and the Spleen Dissociation Kit (Miltenyi Biotec; #130-095-926), respectively. Viable cells were identified by staining with propidium iodide (Miltenyi Biotec; #130-093-233) and incubated with the following antibodies from Miltenyi Biotec: CD11b clone REA592, CD11c clone REA754, major histocompatibility complex class II (MHCII) clone M5/114.15.2, CD80 clone 16-10A1, CD83 clone REA304, Gr1 clone RB6-8C5, and F4/80 clone REA126. The fluorescently labeled α-bungarotoxin-AF647 (α-Bgtx-AF647) was obtained from Thermo Fisher Scientific. The specificity of cell staining was determined using fluorescence minus one controls, and all cell staining data acquired with a MACSQuant10 flow cytometer (Miltenyi Biotec), as recommended by the manufacturer. Data were analyzed using MACSQuant V2.11 (Miltenyi Biotec) to gate cell populations and measure percentages, and FlowJo V10 (Becton-Dickinson; RRID: SCR_008520) to determine mean fluorescence intensity and generate histogram overlays and analyses.

### Cell separation and gene expression

Tumor-infiltrating CD45^+^ cells were isolated from primary tumors according to manufacturer’s procedures (Miltenyi Biotec; #130-110-618). Cells were lysed in TRIzol (Thermo Fisher Scientific; #15596018), and RNA prepared using Direct-zol (Zymo; #R2070). Quantitative real-time polymerase chain reactions were used to measure CHRNA7 gene expression using complementary DNA prepared with iScript (Bio-Rad; #1708891), SsoAdvance SYBR green (Bio-Rad; #172-5271), and commercial primers for GAPDH (#QT01658692; Qiagen) and custom primers for CHRNA7 (5′-CAGTGGTCGTGACAGTGATT-3′ and 5′-CCTGGTCCACTTAGGCATTT-3′) (Integrated DNA Technologies). Reactions were performed using a CFX96 (Bio-Rad) for 40 cycles, data acquired with CFX Manager Software 3.1 (Bio-Rad) and analyzed using the 2(−Delta Delta C(T)) method with test genes compared relative to GAPDH.^15^ For NanoString analyses, the myeloid gene panel V2 (University of California San Diego Sanford Stem Cell Consortium Core Facility) was used and analyzed using nCounter Analysis to identify changes in expression levels of myeloid genes from CD45-enriched tumor-infiltrating leukocytes.

### Enzyme-linked immunospot assay

To assess the effect of AR-R17779 treated dendritic cells on CD8^+^ cytotoxic T cells, an enzyme-linked immunospot (ELISpot) assay for interferon γ (IFN-γ) was performed using the Mouse IFN-γ ELISpot Kit (R&D Systems, #EL485) per the manufacturer’s protocol. Briefly, BMDCs were prepared by differentiation of bone marrow isolated by centrifugation of femurs from C57BL/6 mice,^16^ and cultured in granulocyte-macrophage colony-stimulating factor as previously described for 7 d.^17^ BMDCs were then treated with escalating doses of AR-R17779 (0.2 µM, 2 µM, 20 µM) for 24 h. BMDCs were then pulsed with 5 µg/mL of ovalbumin_257–264_ peptide (InvivoGen; #vac-sin) for 18 h. CD8^+^ T cells were then isolated from OT-I mice (C57BL/6-Tg(TcraTcrb)1100Mjb/J) obtained from the Jackson Laboratory (RRID: IMSR_JAX:003831) with the mouse CD8a^+^ T Cell Isolation Kit (Miltenyi Biotec; # 130-104-075) to obtain T cells that express T cell receptors that recognize Ovalbumin_257–264_ (Ova_257–264_).^18^ DCs and T cells were then cocultured in the ELISpot plate precoated with anti-IFN-γ capture antibody (R&D Systems; #890894) at various DC:T cell ratios (1:1, 1:5, 1:10) for 48 h. Detection antibody for IFN-γ (R&D Systems; #890895) was then added to wells and incubated overnight. Streptavidin-AP concentrate (R&D Systems; #895358) was added and incubated at room temperature for 2 h. The BCIP/NBT substrate (R&D Systems; #895867) was added and incubated at room temperature for 1 h. The plate was washed and dried before spot counting with an Immunospot S5 UV Analyzer (Cellular Technology Limited). Comparisons of IFN-γ secretion were made between the positive control and the treated BMDCs (0.2 µM, 2 µM, 20 µM) with the untreated BMDCs. Treatment of BMDCs with lipopolysaccharide (10 ng/mL) was performed as a positive control and untreated OT-I CD8^+^ T cells were used as a negative control.

### CD8^+^ T cell depletion

CD8^+^ T cell depletion was performed in the 4T1 model as previously described^19^ to further elucidate the mechanism of AR-R17779 in activating dendritic cells to promote an antitumorigenic cytotoxic T cell response. Mice bearing MFP tumors were randomized to injection with vehicle, AR-R17779 with anti-CD8 antibody (InVivoMAb anti-Mouse CD8α; Bio X Cell; # BE0004-1; Clone 53-6.7), AR-R17779 with rat IgG2a isotype control antibody (I*nVivo*MAb rat IgG2a isotype control, antitrinitrophenol; Bio X Cell; # BE0089; Clone 2A3), or anti-CD8 antibody alone. Randomization was performed on day 20 post–tumor inoculation to account for tumor volume at the start of treatment. Intraperitoneal injections of anti-CD8 or isotype control antibody (100 µg/200 mL in phosphate-buffered saline) were performed for 3 consecutive days before initiating treatment with AR-R17779 or vehicle followed by a subsequent dose on day 25 post–tumor inoculation. Maintenance injections were performed every 6 d until euthanasia. CD8^+^ T cell depletion was confirmed by flow cytometry.

## Results

### Loss of CHRNA7 in the host is permissive to TNBC progression

**Figure 1.**
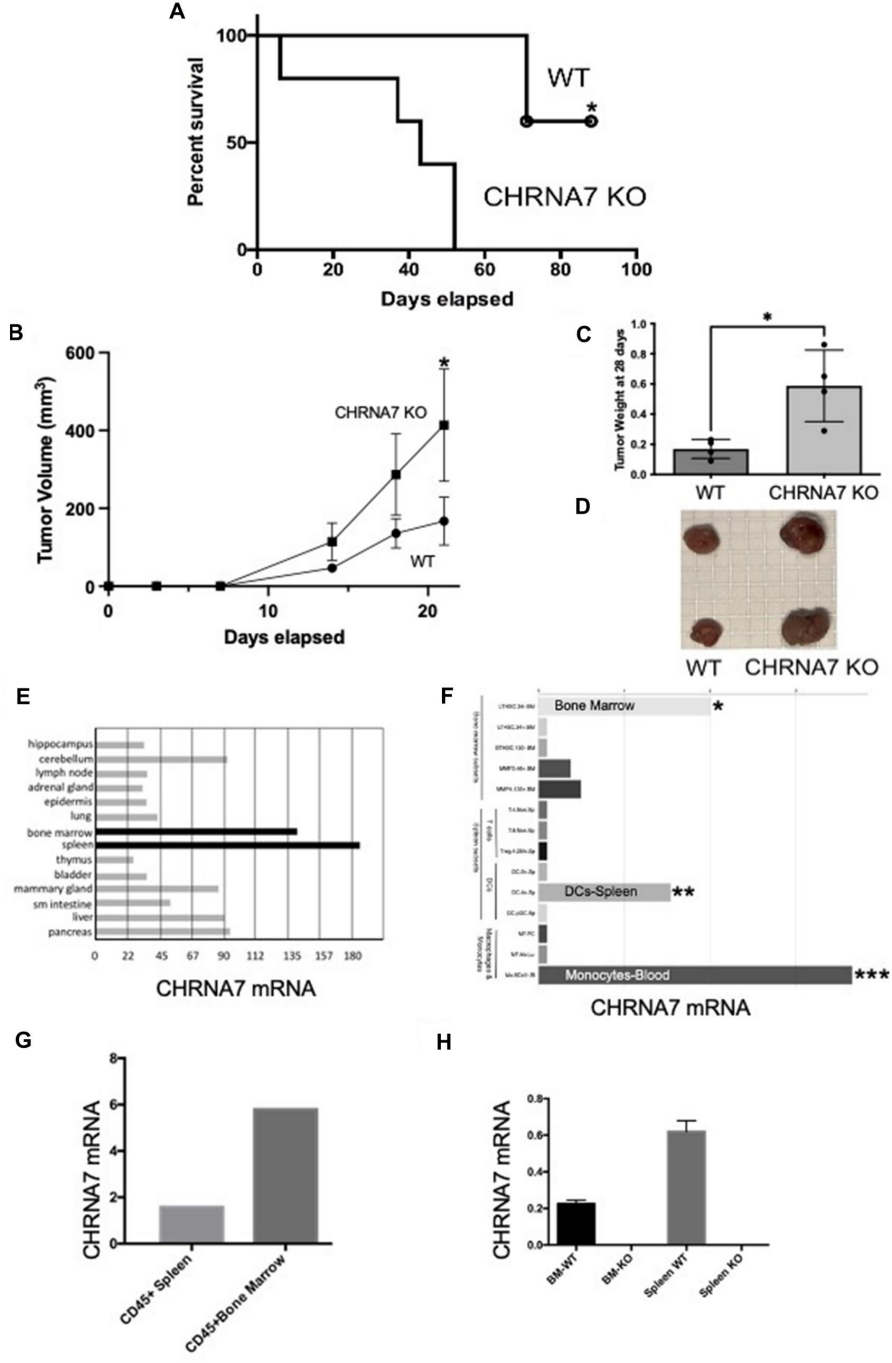
CHRNA7^KO^ mice have decreased survival and increased TNBC growth. (A) CHRNA7^KO^ and WT sibling matched control mice were injected under the fourth MFP with E0771 tumor cells (1 × 10^6^), a mouse model of TNBC syngeneic with the C57BL/6 background of CHRNA7^KO^ mice. (B) Tumor size was monitored for 21 d after injection. (C) Tumor weight was measured at day 28 prior to analysis for tumor-infiltrating immune cells, and (D) representative tumors imaged upon harvest at 28 d. (E) High levels of CHRNA7 gene expression in mouse bone marrow (BM) and spleen. (F) In enriched cell subsets of BM and spleen and in monocytes, CHRNA7 is also highly expressed based on data obtained through the Immunological Genome Project. (G) Gene expression of CHRNA7 in CD45^+^ mouse splenocytes and bone marrow. (H) CHRNA7 expression in CHRNA7^WT^ and CHRNA7^KO^ BM and spleen. The survival curve in (A) is representative of a single experiment performed (n = 6 in each arm). Tumor volumes in (B) are representative of a single experiment performed (n = 7–8 in each arm). The tumor weight plot in (C) is representative of a single experiment performed (n = 4 in each arm). Kaplan-Meier survival analysis with log-rank test in panel A, Two-way analysis of variance in panel B, and Welch’s *t* test in panel C. All data are plotted as mean ± SEM. *P < 0.05; **P < 0.01; ***P < 0.001. mRNA, messenger RNA.

To determine whether CHRNA7 had a functional role in the host compartment of a syngeneic TNBC tumor model, tumor cells were implanted into the MFP of knockout mice that lacked CHRNA7 (CHRNA7^KO^). E0771 tumor cells matched to the C57BL/6 background^13^ were injected into the MFP of CHRNA7^KO^ and CHRNA7^WT^ mice and the effects on overall survival and tumor size determined (Fig. 1). We observed that CHRNA7^KO^ mice had a significant reduction in survival (Fig. 1A), increased primary tumor growth (Fig. 1B), and increased tumor wet weight compared with CHRNA7^WT^ mice (Fig. 1C, D). Based on these genetic findings demonstrating a functional role for CHRNA7 action in host cells, we focused on an analysis of the IMMGEN database, which showed high expression of CHRNA7 in myeloid immune cells, consistent with previous reports showing CHRNA7 expression and activity in macrophages.^8^ At the level of whole tissues, bone marrow and spleen had high levels of CHRNA7 expression (Fig. 1E, black bars), with isolated blood monocytes, splenic DCs, and hematopoietic stem cells being the major sources of CHRNA7 in normal mice (Fig. 1F, labeled bars). We validated the relatively higher levels of CHRNA7 gene expression in CD45^+^ cells enriched by magnetic beads, followed by quantitative polymerase chain reaction analysis of CHRNA7^KO^ versus CHRNA7^WT^ bone marrow and spleen to demonstrate assay specificity (Fig. 1G, H). Together, these observations supported a functional role for CHRNA7 in mediating tumor progression, which along with increased CHRNA7 expression in myeloid immune cells, suggesting that CHRNA7 may be a novel therapeutic target.

### CHRNA7^KO^ leads to immune cell changes in blood, spleen, and tumor-associated cells

**Figure 2.**
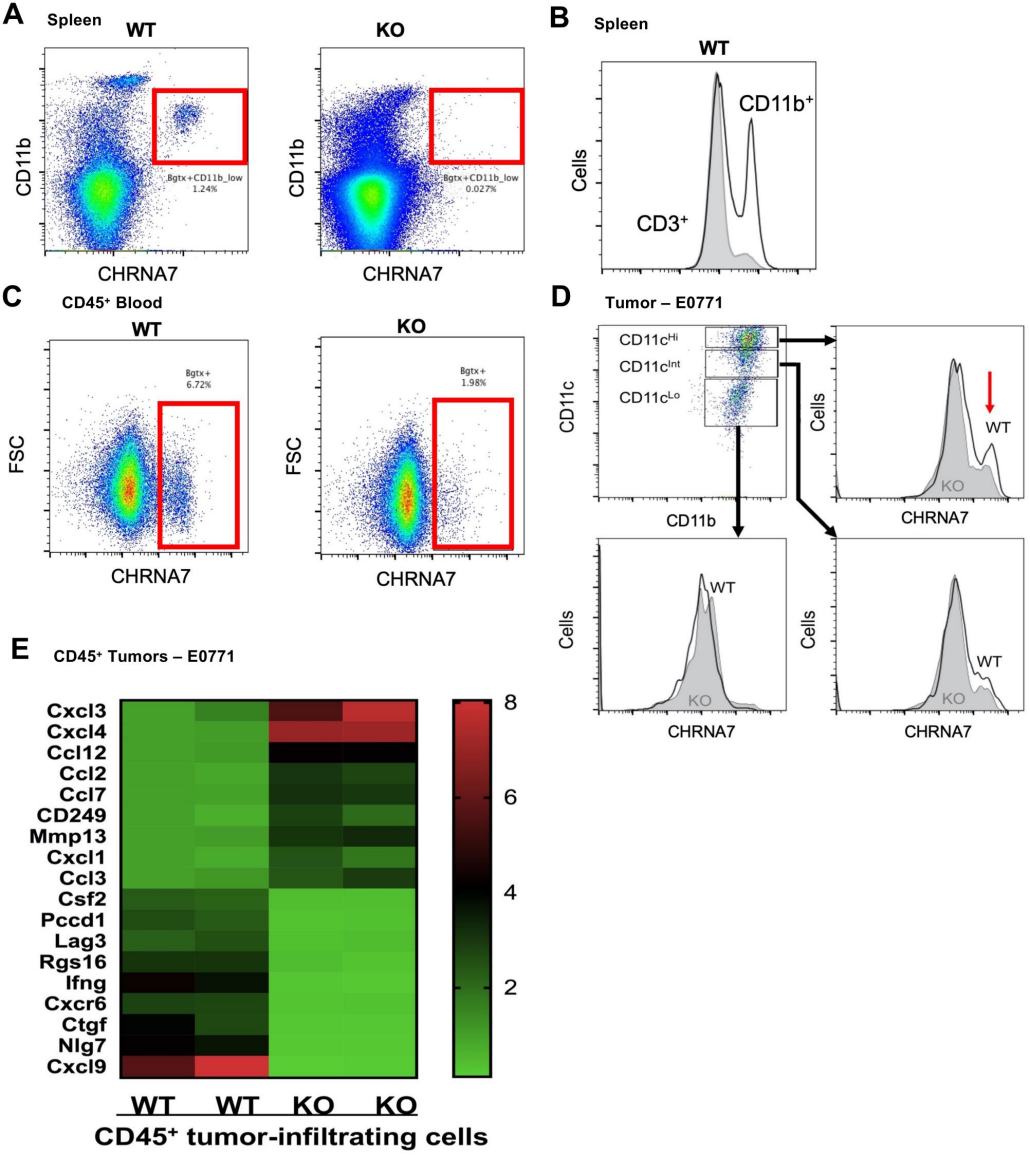
CHRNA7 protein distribution and pathway analysis of tumor-associated leukocytes from CHRNA7^WT^ and CHRNA7^KO^ mice. (A) CHRNA7 protein was detected in CHRNA7^WT^ splenocytes by flow cytometry using the CHRNA7-specific probe, fluorescent α-bungarotoxin (α-Bgtx), and compared with CHRNA7^KO^ to establish gating. (B) Overlay of CHRNA7 expression in CD11b^+^ myeloid versus CD3^+^ lymphoid splenocytes based on α-Bgtx staining to establish the relative abundance of CHRNA7 protein in CD45^+^ immune cell pools. (C) Expression of CHRNA7 protein from CD45^+^ leukocytes from the blood based on α-Bgtx staining. (D) Intratumoral (E0771) expression of CHRNA7 determined using α-Bgtx staining of CD11b^+^ cells stratified based on CD11c levels to identify DC populations (top right, red arrow). (E) NanoString analysis of gene expression of E0771 tumor-associated CD45^+^ leukocytes to define a CHRNA7-regulated myeloid gene expression signature.

We next assessed CHRNA7 protein levels in spleen and peripheral blood leukocytes and analyzed intratumoral expression of CHRNA7 protein on leukocytes using a fluorescent analog of the snake venom toxin α-Bgtx, a peptide that binds CHRNA7 protein with high specificity.^20^ Using α-Bgtx to detect CHRNA7 by flow cytometry, we identified a population of CD11b^+^ splenocytes expressing CHRNA7 protein, with CHRNA7^KO^ cells being used as a negative control for specificity (Fig. 2A). An overlay of α-Bgtx staining of CD11b^+^ cells over α-Bgtx staining of CD3^+^ lymphoid splenocytes revealed a lower level of staining in CD3^+^ lymphoid cells relative to higher CHRNA7 levels on CD11b^+^ myeloid cells (Fig. 2B, overlay), consistent with the higher levels of CHRNA7 gene expression shown previously (Fig. 1E–H). Similarly, staining CD45^+^ peripheral blood leukocytes with fluorescent α-Bgtx identified CHRNA7^+^ leukocytes in the circulation of tumor-bearing mice (Fig. 2C). To further assess the relative distribution of CHRNA7 in E0771 tumors using α-Bgtx staining, cells were analyzed by flow cytometry based on CD11b^+^ and CD11c^+^ immunostaining (Fig. 2D). We observed that the highest levels of CHRNA7 expression were observed on CD11c^Hi^CD11b^+^ tumor-associated cells (Fig. 2D, top right, red arrow), a myeloid cell subset associated in general with APCs. Using CHRNA7^KO^ cells to gate for specificity of α-Bgtx staining, we observed low to no detectable levels of CHRNA7 protein on CD11c^Int^ or CD11c^Lo^cells (Fig. 2D, bottom row). Based on the expression of CHRNA7 in CD45^+^ cells of spleen, peripheral blood and tumors, and the role of CHRNA7 in transcriptional signaling,^9^ we isolated tumor-associated CD45^+^ cells by magnetic beads from the primary E0771 tumors of CHRNA7^KO^ and CHRNA7^WT^ mice and performed a NanoString immune cell–related gene panel analysis (Fig. 2E). This approach identified an increase in CXCL3, CXCL4, and CCL2 in CHRNA7^KO^ cells and a decrease in CSF2, Pccd1, IFNγ, CXCR6, and CXCL9. Together, these changes suggested that CHRNA7 mediated a reprogramming of gene expression in tumor-associated immune cells that was relevant in tumor progression. For example, decreased CXCL9 suggested that a reduction in CD8^+^ CHRNA7^KO^ tumor-infiltrating CD8^+^ lymphocytes (TILs) may be a recruitment defect, while increased CCL2 could be related to the recruitment of myeloid-derived suppressor cells.^21^^,^^22^

### CHRNA7^WT^ immune cells have a distinct gene expression profile compared with CHRNA7^KO^

**Figure 3.**
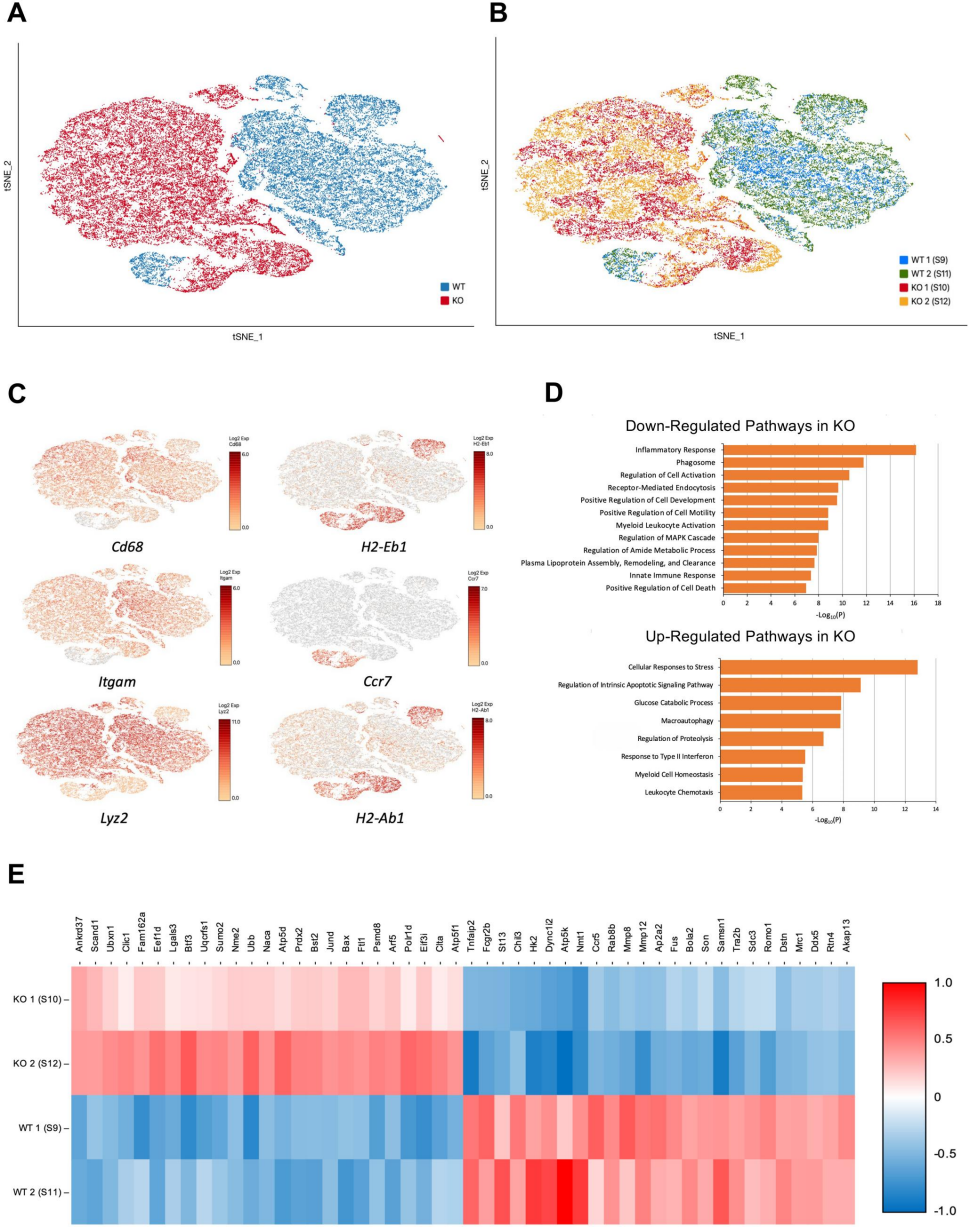
Single-cell transcriptomic analysis of CHRNA7-regulated gene expression in BMDCs. (A) scRNAseq analysis of BMDCs identifies distinct populations from pooled BMDCs of CHRNA7^WT^ versus CHRNA7^KO^ mice. (B) Analysis of individual replicates of CHRNA7^WT^ versus CHRNA7^KO^ mice. (C) Identification of genes relevant to BMDC differentiation. (D) Pathway analysis of genes down/upregulated in CHRNA7^KO^ mice compared with control CHRNA7^WT^ BMDCs. (E) Top genes down/upregulated in BMDCs from CHRNA7^WT^ versus CHRNA7^KO^ BMDCs.

To determine whether CHRNA7-mediated changes in the in vivo immune cell transcriptional profile of the intact tumor were reflected in an in vitro cell autonomous transcriptional analysis, we performed single-cell RNA sequencing (scRNAseq) on cell types relevant to the α-Bgtx^+^CD11c^+^CD11b^+^ cells described previously. We established primary cultures of bone marrow–derived myeloid cells following differentiation with macrophage colony-stimulating factor and IL-4, which yields a mix of macrophages and dendritic cells (bone marrow–derived macrophages [BMDMs]/BMDCs). Using BMDMs/BMDCs to define a role for CHRNA7-mediated signaling, we performed a principal component analysis and observed distinct cell clustering of CHRNA7^KO^ (Fig. 3A) versus CHRNA7^WT^ BMDMs/BMDCs based on gene signatures (Fig. 3A). These distinct clusters were defined by changes in pathways related to the tumor-immune microenvironment. The substantial differences in gene expression between CHRNA7^KO^ and CHRNA7^WT^ (Fig. 3A) were consistent upon analysis of independent biological replicates of CHRNA7^KO^ (Fig. 3B) versus CHRNA7^WT^ (Fig. 3B) cells. Analysis of the distribution of classic expression markers for macrophages and DCs showed a distribution of cell types based on expression of macrophage markers (i.e. CD68, CD11b/ITGAM, Lyz2) versus DC markers (i.e. CCR7, H2-Eb1, and H2-Ab1) (Fig. 3C). Pathway analysis based on DAVID (david.ncifcrf.gov) identified pathways regulating inflammatory responses, cell activation, and leukocyte migration that were downregulated in CHRNA7^KO^ BMDMs/BMDCs (Fig. 3D, top) and pathways that were upregulated in CHRNA7^KO^ BMDMs/BMDCs such as stress responses, apoptotic signaling, catabolic processes, and macroautophagy (Fig. 3D, bottom) compared with CHRNA7^WT^ cells. At an individual gene level, we identified increases in gene expression in CHRNA7^KO^ versus CHRNA7^WT^ BMDMs/BMDCs of Ankrd37 that was associated with hypoxia and Scand1 with transcriptional regulation, and Ubx1, Sumo2, and Ubb that were associated with regulation of ubiquitination (Fig. 3E). In contrast, CHRNA7^WT^ BMDMs/BMDCs were characterized by increases in cells expressing tumor necrosis factor α–induced protein 2 ((TNFaip2) that regulated TNFα signaling, macrophage markers like the Fcγ receptor (Fcgr2b), a chemokine receptor (CCR5), matrix proteases (MMP8 and MMP12), and mannose receptor (Mrc) (Fig. 3E). Together, these findings supported a role for CHRNA7 in regulating gene expression in BMDMs/BMDCs.

### CHRNA^KO^ reduces immune surveillance markers in the tumor microenvironment

**Figure 4.**
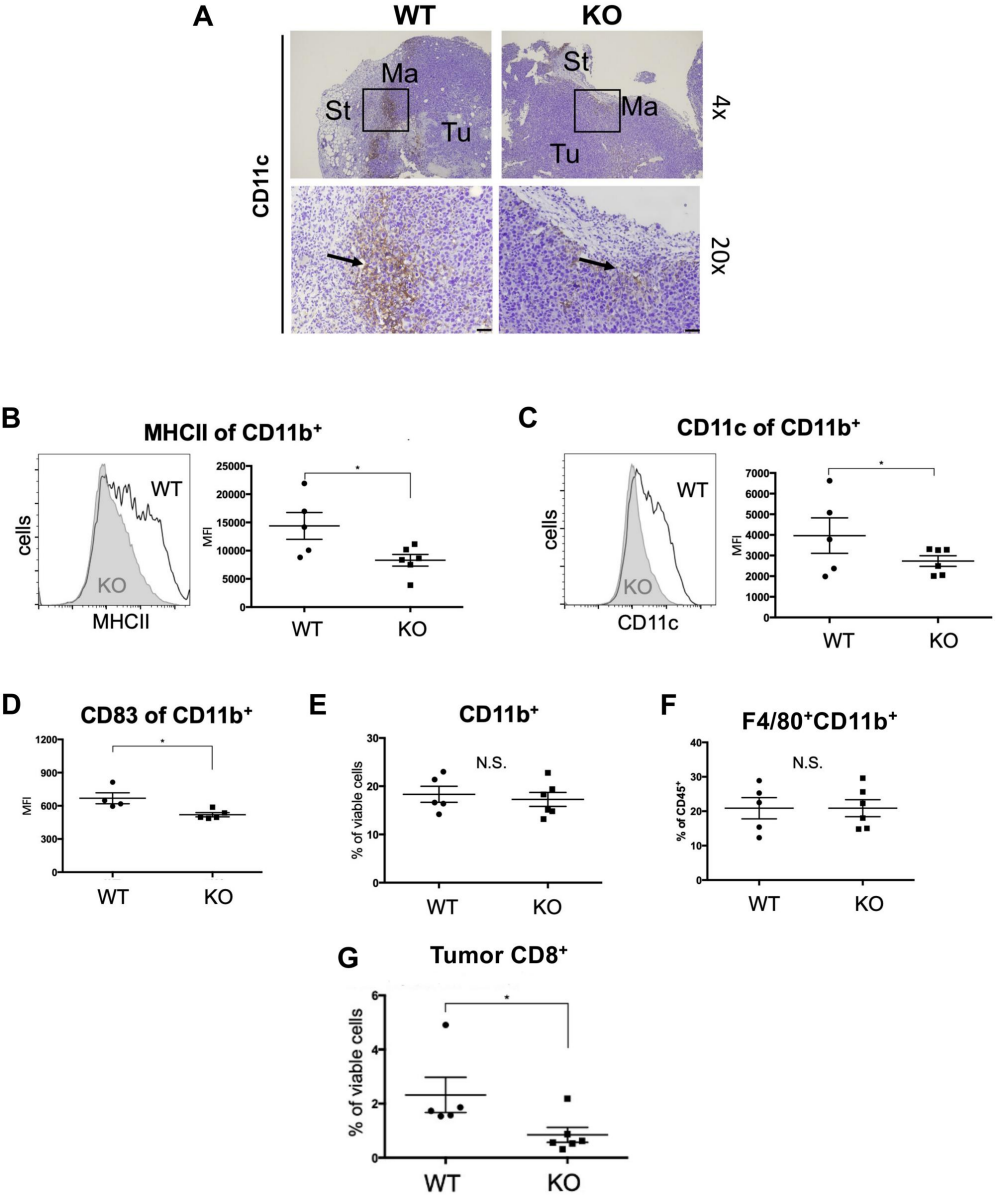
Analysis of immune markers associated with tumor immune surveillance in tumor-bearing CHRNA7^KO^ mice. (A) Immunolocalization of CD11c^+^ cells in the tumor margins of CHRNA7^WT^ versus CHRNA7^KO^ mice of primary E0771 tumors injected into the MFP. (B) Quantification of CHRNA7-mediated reductions in MHCII levels on subsets of tumor-associated CD11b^+^ cells in CHRNA7^KO^ mice. (C) Analysis of CD11c^+^CD11b^+^ subsets in tumors. (D) Analysis of CD83^+^CD11b^+^ cells. (E) Analysis of CD11b^+^ cells and (F) F4/80^+^CD11b^+^ (not significant). (G) Analysis of CD8^+^ cells. Individual dots are representative of technical replicates from a single experiment (n = 4–6 in each arm). Student’s *t* test in panels B to G. Data are shown as mean fluorescence intensity ± SD. *P < 0.05; N.S., not significant; Ma, margin; St, stroma; Tu, tumor.

With in vitro data supporting a functional role for CHRNA7 activity in immune cell activation (Fig. 3), and the tumor permissive phenotype of CHRNA7^KO^ mice (Fig. 1), we performed a combination of immunohistochemistry and flow cytometry to localize and quantify tumor-associated immune cells. To localize CHRNA7-dependent changes in tumor-associated immune cells, we immunostained E0771 tumors from CHRNA7^KO^ versus WT mice with an anti-CD11c antibody (Fig. 4). We identified CD11c^+^ cells localized in the tumor margins (Fig. 4A, left) of WT mice, while CHRNA7^KO^ mice lacked CD11c^+^ cells in representative tumor sections (Fig. 4A, right). Flow cytometry was performed to identify changes in the immune cell profile between CHRNA7^KO^ and WT mice. CHRNA7^KO^ mice had decreased levels of MHCII, CD11c, and CD83 expression in CD11b^+^ cell subsets (Fig. 4B–D), while no overall changes in the numbers of CD11b^+^ or F4/80^+^ tumor macrophages were observed (Fig. 4E, F). Based on the reduced expression of APC activation markers such as MHCII, CD11c, and CD83 on CD11b^+^ cells, we analyzed tumors for changes in the number of tumor-associated CD8 T cells and observed a decrease in TILs in CHRNA7^KO^ mice compared with WT mice (Fig. 4G). These findings suggested a link between increased tumor burden in CHRNA7^KO^ (Fig. 1) and reduced expression of APC activation markers and recruitment of CD8^+^ TILs in vivo (Fig. 4).

### In vivo activation of CHRNA7 reduces tumor burden and increases survival in TNBC

**Figure 5.**
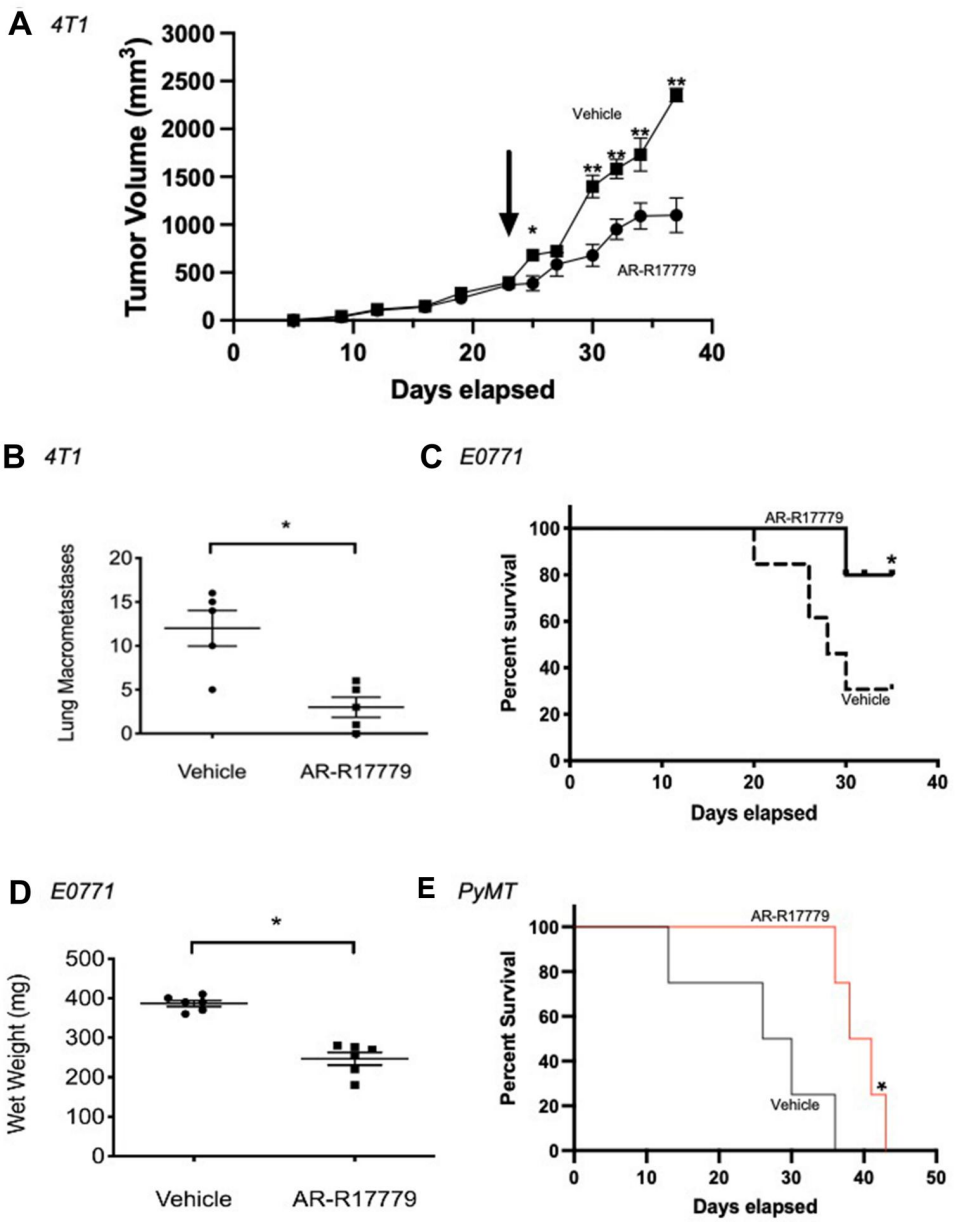
Pharmacological testing of the CHRNA7 agonist AR-R17779 in mouse models of TNBC. (A) Tumor volume of syngeneic BALB/c mice injected with 4T1 mammary carcinoma cells, following treatment with vehicle versus AR-R17779 (1 mg/kg, intraperitoneal [IP], every 2 d) starting on day 23 (black arrow). (B) Quantification of lung macro metastases from vehicle-treated versus AR-R17779–treated mice in the 4T1 tumor model. (C) Survival of a syngeneic C57BL/6-based tumor model using E0771 tumor cells matched to the genetic background of CHRNA7^KO^ mice following treatment with vehicle versus AR-R17779 (1 mg/kg, IP, every 2 d). (D) Reduced primary tumor wet weight of AR-R17779–treated mice compared with vehicle. (E) Survival of the spontaneous MMTV-PyMT: FVB mammary tumor model following treatment with vehicle versus AR-R17779 (1 mg/kg, IP, every 2 d). Tumor volumes in (A) are technical replicates of 1 representative experiment, out of 3 performed (n = 5–8 in each arm). Lung macrometastases in (B) are technical replicates of 1 representative experiment, out of 2 performed (n = 5–6 per arm). The survival curves in (C) and (E) are representative of a single experiment performed (n = 6 in each arm). The tumor weight plot in (D) is representative of a single experiment performed (n = 6 in each arm). Two-way analysis of variance with Holm-Sidak correction in panel A, Welch’s *t* test in panels B and D, and Kaplan-Meier survival analysis with log-rank test in panels C and E. All tumor volume data are plotted as mean ± SE. Wet weight and lung macrometastases data are presented as mean ± SD. *P < 0.05; **P < 0.01.

Based on genetic approaches establishing a tumor-permissive phenotype using CHRNA7^KO^ mice (Fig. 1), we next tested a pharmacological approach that focused on in vivo testing of a small molecule agonist that is highly specific for CHRNA7, AR-R17779, in several different models of TNBC (Fig. 5). First, we used 4T1 mouse carcinoma cells that were syngeneic and highly metastatic to the lung in BALB/c mice to test the effect of AR-R17779 on tumor progression. Mice were injected with 4T1 tumor cells, monitored for 23 d, and then randomized for intraperitoneal administration of AR-R17779 every 48 h. Reductions in tumor volume (Fig. 5A) were observed following treatment with AR-R17779 compared with vehicle control with significant reductions in tumor volume in the AR-R17779–treated group from days 25 through to tumor harvest on day 37 (AR-R17779 = 1,099 mm^3^; vehicle = 2,355 mm^3^; *P* = 0.0046). To assess the effects of AR-R17779 on lung metastases, we injected a separate cohort of mice with 4T1 tumor cells that were monitored for 10 d, and the primary tumor resected, as detailed in the Materials and Methods. Mice were then randomized and administered AR-R17779 or vehicle control for an additional 10 d, at which point lungs were harvested for enumeration of lung macrometastases. Significant reductions in metastases were observed in AR-R17779–treated mice vs controls (*P* = 0.0363) (Fig. 5B). To compare the antitumorigenic effects of AR-R17779 in a second immune competent breast tumor model, we injected E0771 tumor cells into C57BL/6 mice that were then treated with AR-R17779 vs vehicle control by intraperitoneal injection every 48 h. In the AR-R17779–treated group, we observed a significant increase in overall survival (Fig. 5C) and decreased tumor wet weight (Fig. 5D). In a third mouse model of immune competent breast cancer, we tested the effects of AR-17779 on tumor progression in a genetic model of spontaneous onset of breast cancer in transgenic mice expressing Polyoma Middle T under the control of the MMTV promoter (PyMT-MMTV). This multifocal TNBC tumor model in the FVB strain was subjected to treatment with AR-R17779 versus vehicle control every 48 h, in which we observed a significant increase in survival in AR-R17779–treated mice compared with vehicle-treated mice (Fig. 5E). Together, these findings show that AR-R17779 has an antitumorigenic effect in multiple models of immune-competent breast cancer that led us to consider whether this therapeutic approach was relevant to our studies on the expression of CHRNA7 observed in myeloid immune cells from the previous studies.

### Treatment effect of AR-R17779 synergizes in combination with anti-PD-L1 therapy

**Figure 6.**
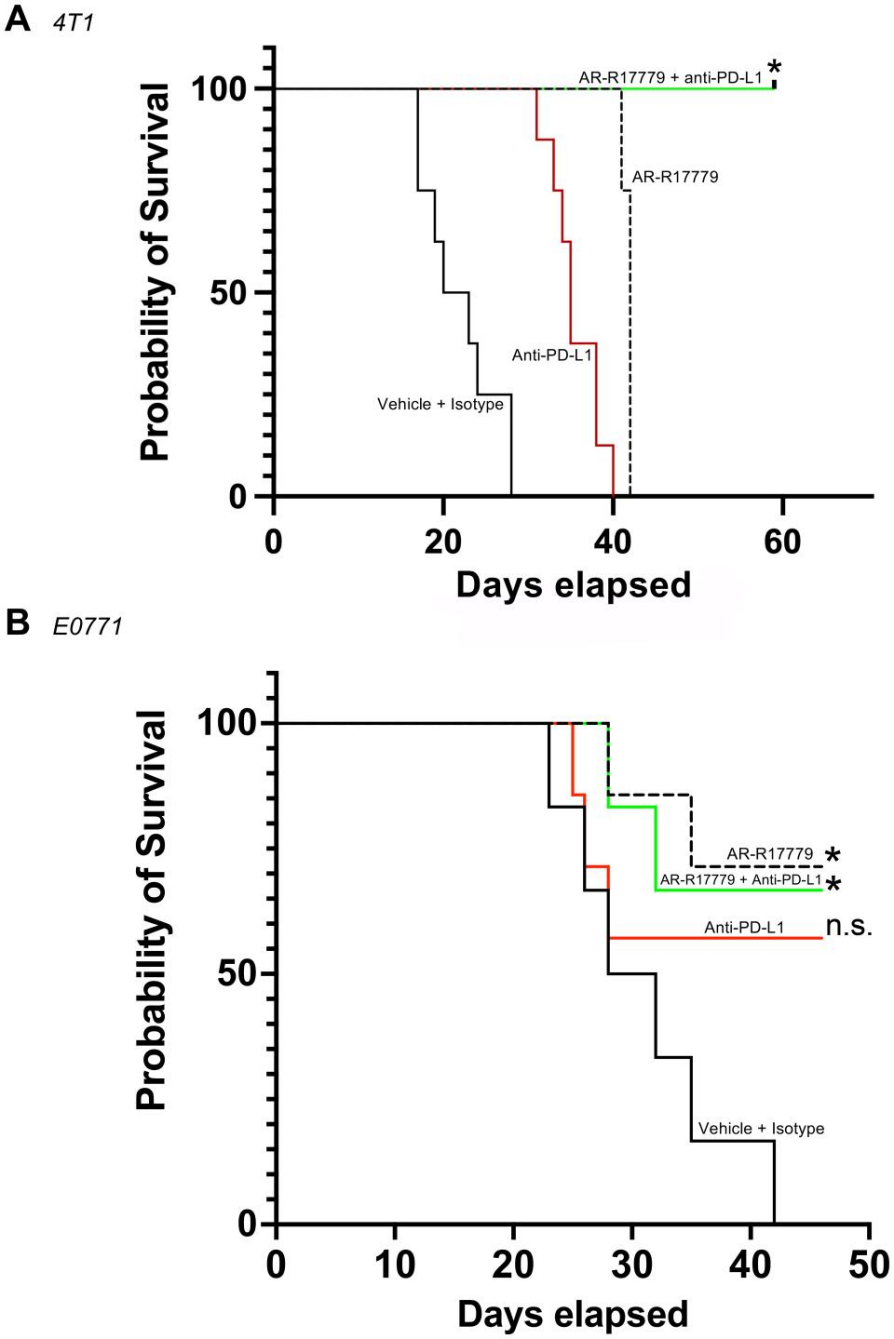
Effect of monotherapy versus combination therapy on mouse survival. (A) Survival of mice in the 4T1/BALB/c mouse model subjected to treatment with monotherapy with AR-R17779 or anti-PD-L1 versus combination therapy of anti-PD-L1 with AR-R17779 compared with a vehicle control. (B) Survival of mice in the E0771/C57BL/6 mouse model subjected to treatment with monotherapy with AR-R17779 or anti-PD-L1 versus combination therapy of anti-PD-L1 with AR-R17779 compared with vehicle control. The survival curve in (A) is representative of 1 experiment performed (n = 8 in each arm). The survival curve in (B) is representative of 1 experiment, out of 2 performed (n = 7 in each arm). Kaplan-Meier survival analysis with log-rank test in panels A and B. *P < 0.05. n.s., not significant.

Based on the efficacy of AR-R17779 treatment on reducing tumor progression in several models of TNBC, and evidence that CHRNA7 action in tumor-associated immune cells is relevant in adaptive immunity, we tested the effect of combination therapy of AR-R17779 with an anti-PD-L1 antibody (Fig. 6A). We observed that combination treatment with AR-R17779 and anti-PD-L1 led to a significant increase in survival, compared with vehicle control and treatment with either agent alone. To further validate the effect of combination therapy on survival, the experiment was repeated in the E0771 model. We observed that treatment with AR-R17779 monotherapy and combination therapy of AR-R17779 with anti-PD-L1 led to a significant increase in survival, compared with vehicle control (Fig. 6B). Interestingly, while both experiments suggested synergy between AR-R17779 and anti-PD-L1 when used in combination, anti-PD-L1 monotherapy had a beneficial trend in the E0771 model that became significant in a combination therapy with AR-R17779.

### Treatment of BMDCs with AR-17779 increases IFN-γ secretion from OT-I T cells

**Figure 7.**
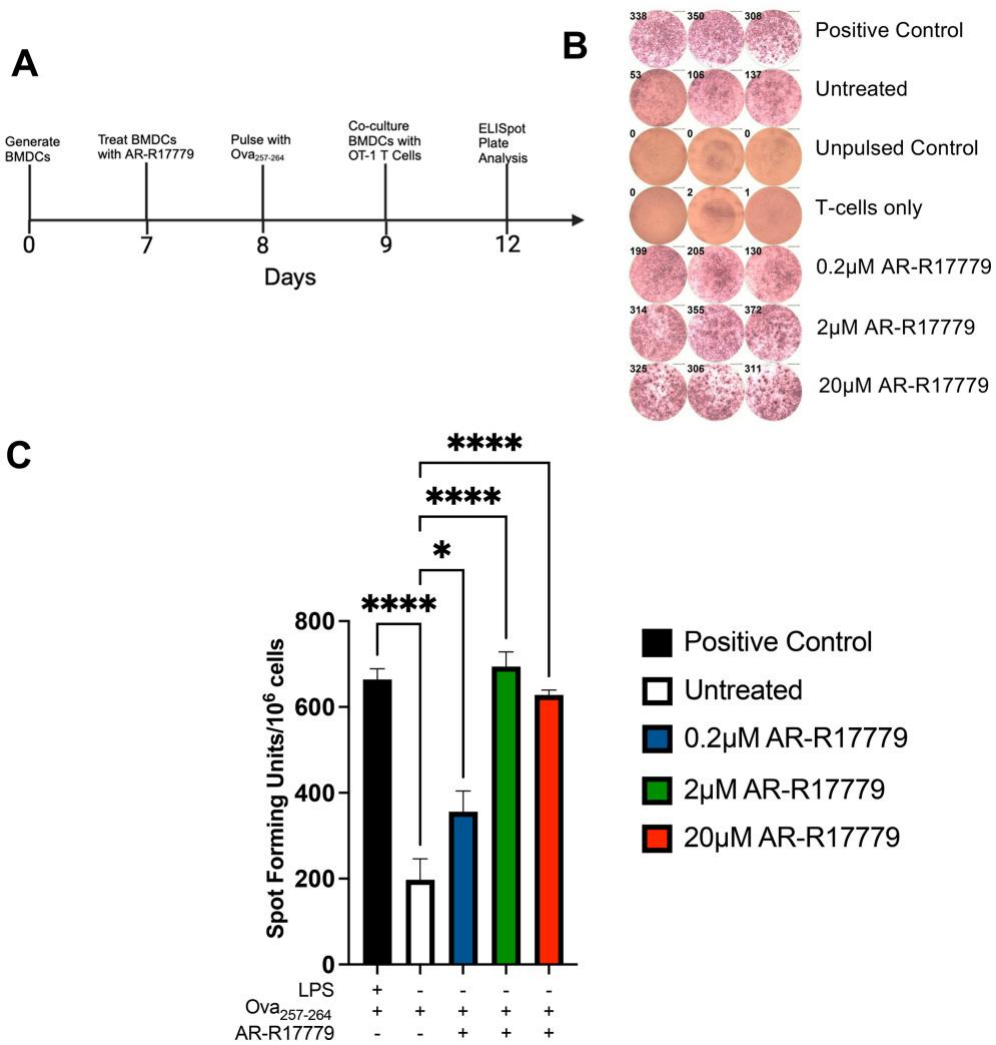
AR-R17779 treated BMDCs increase T cell activation as shown by IFN-γ ELISpot. BMDCs were generated from C57BL/6 mice and treated with AR-R17779 before pulsing with Ova_257–264_. T cells were isolated from OT-I mice via magnetic bead separation. BMDCs and T cells were cocultured on the ELISpot plate for 48 h before plate preparation and reading. (A) Experimental timeline. (B) Representative image from ELISpot plate highlighting experimental conditions and raw spot counts. (C) AR-R17779 increases IFN-γ secretion from OT-I T cells. Spot-forming units are representative of a single experiment (n = 3 per condition). One-way analysis of variance with Holm-Sidak correction in panel C. Data are shown as mean spot-forming units/10^6^ cells ± SEM. *P < 0.05; ****P < 0.0001. LPS, lipopolysaccharide.

To further explore the effects of AR-R17779 on T cell activation, an ELISpot assay was performed for IFN-γ. BMDCs from C57BL/6 mice were generated as previously described^17^ and treated with increasing doses of AR-R17779 before pulsing with Ova_257–264_ and coculture with splenic T cells from transgenic mice (OT-I) expressing T cell receptors specific to Ova_257–264_ (Fig. 7A). IFN-γ secretion was measured by counting spots with an ELISpot reader and normalizing the counts to spot forming units per 10^6^ T cells (Fig. 7B). We observed a significant increase in IFN-γ secretion in the AR-R17779 treated wells compared with the untreated BMDCs (Fig. 7C).

### CD8^+^ T cell depletion uncouples treatment effects of AR-R17779

**Figure 8.**
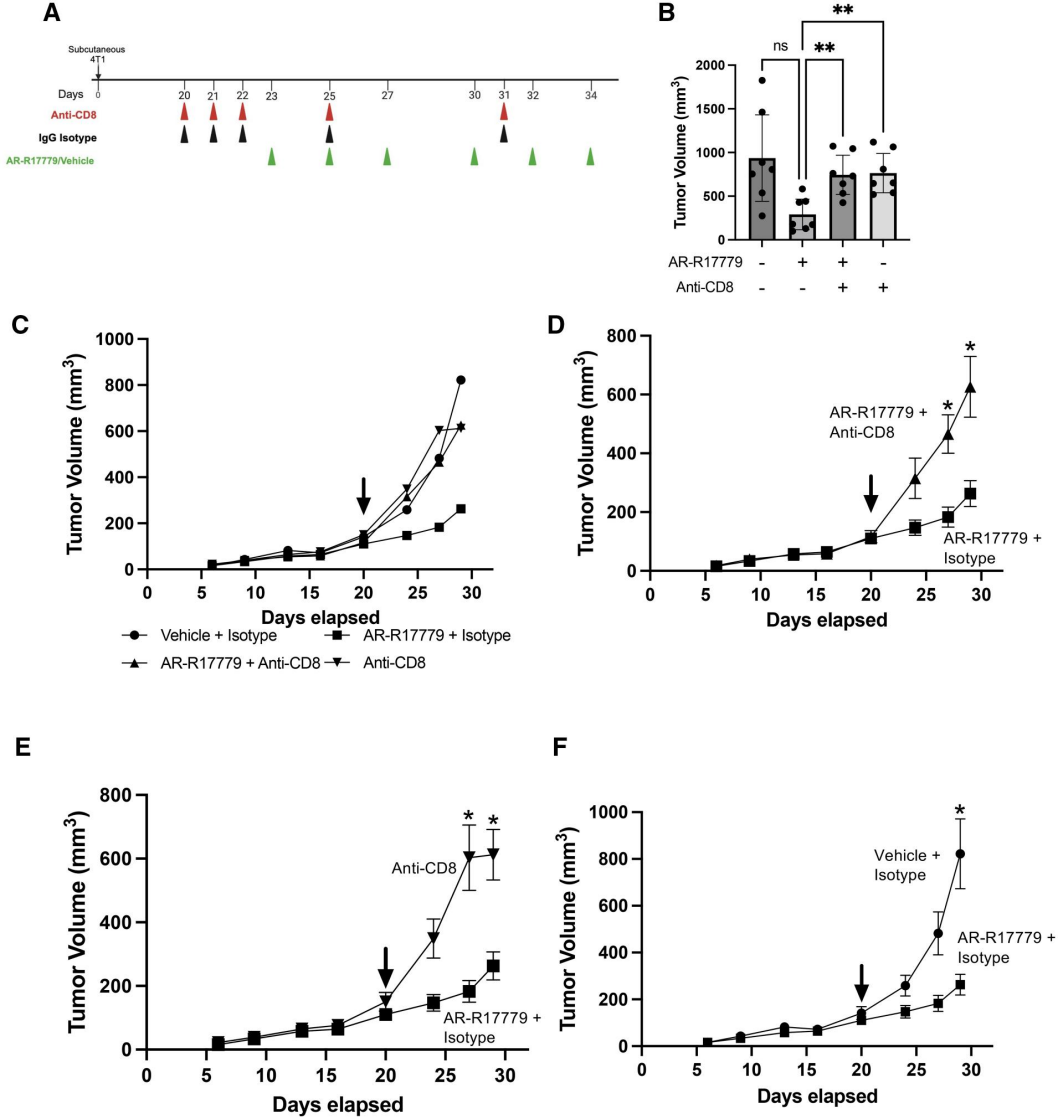
CD8 depletion uncouples antitumor effect of AR-R17779 on 4T1 TNBC tumors. (A) Experimental timeline. Mice were randomized to treatment groups on day 20 after tumor injection. Anti-CD8 injection or anti-IgG Isotype (100 µg/200 µL phosphate-buffered saline) was performed on 3 consecutive days starting on day 20 and treatment with AR-R17779 or vehicle was initiated on day 23 (black arrows in panels C–F). Repeat anti-CD8 or anti-IgG isotype injections were repeated every 6 d. (B) The tumor volume at euthanasia was recorded for each group. (C) Tumor growth curves of each group without comparisons for clarity. (D) Comparison of AR-R17779 and isotype versus AR-R17779 and anti-CD8. (E) Comparison of AR-R17779 and isotype versus anti-CD8. (F) Comparison of AR-R17779 and isotype versus vehicle and isotype. Panels B–F are representative of a single experiment, out of 2 performed (n = 7 per arm). Brown-Forsythe and Welch analysis of variance tests with Dunnett’s T3 test in panel B. Mixed-effects analysis with Holm-Sidak correction in panels C to F. All data are presented as mean tumor volume ± SEM. *P < 0.05; **P < 0.01. ns, not significant.

To determine whether there was a dependence of the antitumorigenic activity of AR-R17779 on CD8 T cells, we tested the in vivo effect of CD8 depletion in the 4T1 tumor. 4T1 tumor cells were injected into the MFP as previously described and tumors monitored until day 20 before randomization and treatment (Fig. 8A). Tumor volume measurements showed that CD8 depletion uncoupled the effect of AR-R17779 treatment in the AR-R17779 + anti-CD8 group, as evidenced by the increased tumor volume after treatment with anti-CD8 antibody (Fig. 8B). Treatment of 4T1 tumors with AR-R17779 alone led to a reduction in tumor growth compared with treatment with AR-R17779 + anti-CD8 antibody (Fig. 8D), anti-CD8 antibody alone (Fig. 8E), and vehicle with isotype (Fig. 8F). These results highlighted the potential of AR-R17779 as a novel immune modulator based on the dependence of AR-R17779 activity on CD8^+^ T cells.

## Discussion

In this study, we provide the first experimental evidence for CHRNA7 as a biochemical target in activating adaptive immune responses to reduce tumor burden and improve overall survival in immune competent murine breast cancer models. We propose that activation of CHRNA7 drives downstream signaling mediated by transcription factors to reprogram APCs to stimulate adaptive immune responses that convert immunologically “cold” tumors to “hot” tumors. We show that loss of CHRNA7 in KO mice increased tumor burden and reduced animal survival that was associated with decreased numbers of DCs. Supported by data demonstrating that tumor-associated CD11c^+^ immune cells highly expressed CHRNA7, we propose that CHRNA7 is a novel biochemical target for activating tumor-associated DCs that promote adaptive immune responses. Based on genetic approaches that established a functional role for CHRNA7 in the host, we tested the effect of a pharmacological agonist of CHRNA7 that has been validated in the fields of inflammation and neurobehavior. The reduction in tumor burden and increased survival that we observed using the CHRNA7 agonist AR-R17779 in combination with an immune checkpoint inhibitor suggested that tumor-associated CHRNA7^+^ immune cells are novel therapeutic targets.^23^^,^^24^

In the complex milieu of the tumor microenvironment, recent studies have focused on the identification of pathways that reduce immunosuppressive mechanisms that are often present in cancer patients and animal models.^25^ For example, there has been a substantial increase in cancer immunotherapy trials that have yielded mixed results in many cancer types,^26^ but there remains an unmet need to better understand mechanisms of immunosuppression in refractory cancer types such as TNBC.^27^ While inhibition of cytotoxic T lymphocyte activation-4 (CTLA-4) and programmed death-1 (PD-1) on T cells are central to reversing immunosuppression in tumors, tumor-associated myeloid cells such as myeloid-derived suppressor cells, tumor-associated macrophages, and DCs are also involved in suppressing antitumor immunity. With interactions between each of these cell types with each other, tumor cells, T cells, and stromal cells conditioning the inflammatory state of the tumor microenvironment, we focused on CHRNA7 based on its expression on tumor-associated myeloid cells and its role in regulating inflammatory responses in other models. However, this phenotype is complex and poorly understood. For example, recent reports show that treatment with immune checkpoint inhibitors that increase CD8 T cell recruitment was associated with increased CCL2, IFNγ, and CSF2,^28^ however, the increases in IFN-γ and CSF2 we observed in WT mice contrasted with the decreased levels of CCL2 observed. As a chemoattractant for monocytes and macrophages in general, the role of CHRNA7 in the regulation of myeloid cell subtypes will require further study. In another example, the downregulation of CXCL9 in tumor-permissive CHRNA7^KO^ mice was consistent with its upregulation by IFN-γ,^29^ highlighting the importance of CXCL9 in attracting T cells.^30^ Our scRNAseq study identified cell autonomous CHRNA7-mediated changes in gene expression focused on the analysis of BMDMs because we had observed higher levels of CHRNA7 expression in DCs based on α-Bgtx staining. We observed that genes involved in ubiquitination (i.e. Ubx1, Sumo2, and Ubb) and relevant in protein degradation were upregulated in CHRNA7^KO^ BMDCs. Interestingly, recent reports have shown that ubiquitination pathways can regulate pattern recognition receptor signaling, DC maturation required for initiation of adaptive immune responses, and retention of MHCII-peptide complexes in DCs^31^^,^^32^ that we associate here with a tumor-permissive phenotype in CHRNA7 KO mice. In contrast, CHRNA7^KO^ BMDCs downregulated factors associated with DC activation such as CCR5, a chemotactic chemokine receptor expressed on DCs and other immune cells. For the genes that we identified as being downregulated in CHRNA7^KO^ DCs, several others were associated with macrophage function, such as decreases in genes encoding mannose receptor (Mrc) (CD206), Fcγ receptor, matrix metalloproteases (MMP8 and MMP12), and TNFaip2. With TNFaip2 as a primary response gene of TNFα and highly expressed in immune cells, the decrease of TNFaip2 in CHRNA7^KO^ BMDCs could be associated with a loss of responsiveness to inflammatory molecules in the tumor microenvironment of a CHRNA7^KO^ host.^33^ Together, the identification of CHRNA7-regulated chemokines (i.e. NanoString array) and genes (i.e. scRNAseq) established candidate molecular endpoints to better define the role(s) of CHRNA7 in specific myeloid immune cell types involved in adaptive immune responses relevant in tumor progression.

Our findings build upon studies of CHRNA7^KO^ mice in models of infection and injury in which the loss of CHRNA7 leads to unattenuated inflammatory responses^8^^,^^9^^,^^34^ that have been primarily attributed to macrophages. We identified an unexpected role for CHRNA7 in cancer in the regulation of tumor-associated immune cells and activation of adaptive immune responses. In models of injury and endotoxemia, the loss of CHRNA7 increases inflammatory responses by uncoupling the protective effects of innervation by the parasympathetic nervous system such as by the vagus nerve.^34^ The attenuation of inflammatory responses mediated by such nervous system inputs is termed the reflex control of immunity,^35^ and is dependent on CHRNA7 action in myeloid-derived bone marrow progenitors rather than cell-autonomous effects in T cells.^36^ Interestingly, however, T cells are known to synthesize acetylcholine, which stimulates CHRNA7 to complete a vagus nerve circuit that controls innate immune responses in endotoxemia.^37^ While reports that the stimulation of the vagus nerve decreased breast cancer metastasis, the effect on the immune system remains unknown.^38^ Interestingly, there are numerous studies investigating the efficacy of DC-based therapies, albeit through a different mechanism. These studies focus on the classical DC subtype 1 (cDC1) that have a more antitumorigenic phenotype than other subtypes and current studies are investigating the modulation of TIM3, CD40, and STAT3 signaling, among other targets to drive dendritic cells into the cDC1 phenotype to further increase the efficacy of current immune checkpoint inhibitors.^39^^,^^40^ This work further drives our efforts to validate a small molecule agonist for TNBC such as ours.

The premise that AR-R17779 enhances antigen presentation by dendritic cells and promotes subsequent activation of cytotoxic T cells is supported by our ELISpot assay for IFN-γ (Fig. 7). Moreover, our T cell depletion studies (Fig. 8) demonstrate that the antitumor effects of AR-R17779 are dependent on CD8^+^ lymphocytes, emphasizing the importance of an intact adaptive immune response. Taken together, these findings support a model in which CHRNA7 signaling promotes the activation of myeloid immune cells that, in turn, stimulate T cell mediated tumor clearance, reducing tumor burden and improving survival. This positions CHRNA7 as a promising therapeutic target in cancer immunotherapy.

The concept that CHRNA7 functions in immune cells to modulate anti-inflammatory signaling and transcriptional reprogramming of immune cells has been further studied in adoptive transfer studies of injury and infection.^9^^,^^41^ For example, adoptive transfer of BM from CHRNA7^KO^ mice into WT recipient mice was sufficient to recapitulate the phenotype of standard CHRNA7^KO^ mice, with the CHRNA7-mediated effects on cytokine expression being specific to myeloid versus lymphoid cells.^42^ In models of atherosclerosis, loss of CHRNA7 led to an increase in C-reactive protein and IL-6, supporting an anti-inflammatory role for CHRNA7,^43^ while in models of arthritis, loss of CHRNA7 increased TNFα and MCP-1.^44^ Furthermore, the release of acetylcholine from T cells, which stimulates CHRNA7-expressing macrophages,^36^ reinforces the relevance of CHRNA7 signaling and the importance of CHRNA7 action in inflammation and cancer.

AR-R17779 is a conformationally restricted analog of acetylcholine with high specificity for CHRNA7,^5^ and has demonstrated efficacy in preclinical models of neurobehavior, arthritis, and gut injury.^6^^,^^7^^,^^45^ CHRNA7 agonists have also shown protective effects in inflammatory conditions including arthritis,^46^^,^^47^ sepsis,^48^ encephalitis,^49^^,^^50^ and systemic inflammatory response syndrome.^8^ We focused on AR-R17779 in our studies due to its well-characterized subunit specificity (i.e. for α7 versus other subunits) and rigorous structure-function validation.^5^ Based on the impact that the knockout of CHRNA7 had on tumor progression and survival, as well as changes in myeloid immune cell gene expression and tumor infiltration, we chose to perform in vivo testing of AR-R17779 on breast tumor–bearing immune-competent mice. Our findings demonstrate that pharmacologic activation of CHRNA7 reduced tumor progression and improved survival, which led us to explore its effects across multiple models including the E0771 model, the highly aggressive and metastatic 4T1 TNBC model, and the spontaneous multifocal MMTV-PyMT model. These results provided the rationale to investigate the therapeutic synergy between AR-R17779 and immune checkpoint blockade in both E0771 and 4T1 tumors. Subsequently, the 4T1 tumor studies were pursued as an aggressive subtype of TNBC, that readily metastasizes. Given this fact, we were able to explore the effect that AR-R17779 may have on lung metastasis. These findings were subsequently validated in the spontaneous MMTV-PyMT model, prompting us to explore further into immune checkpoint inhibitors in both the 4T1 and E0771 tumor models.

Collectively, these data underscore the importance of understanding the mechanism of action, identifying target immune cell population, and establishing validated readouts to optimize the translational potential of AR-R17779 as a novel therapeutic. This mechanistic insight will be essential for designing clinical strategies that incorporate CHRNA7 agonists into studies.

## Conclusions

The integration of genetic and pharmacologic studies highlighted the role of CHRNA7 in tumor-associated immune modulation and supported the therapeutic potential of CHRNA7 agonists in TNBC. Our findings suggest that AR-R17779 as a monotherapy was effective as a stimulator of CHRNA7 signaling, which activated myeloid immune cells leading to increased T cell–mediated tumor clearance to reduced tumor burden and increased survival. Furthermore, when used in combination with immune checkpoint inhibitors, AR-R17779 further enhanced antitumor immunity. Specifically, combining AR-R17779 with anti-PD-L1 therapy significantly increased survival in models where PD-L1 blockade alone has minimal effect, such as TNBC,^51–53^ an effect that we show here was dependent on CD8^+^ T cells. These results support further preclinical and translational investigation, with the goal of informing clinical trials that incorporate CHRNA7-targeted therapies alongside immune checkpoint inhibitors.

## Data Availability

Single cell RNA sequencing files are available at NCBI (GSE299680). This article does not report original code.

## References

[1] Hanahan D , Weinberg, Robert A. Hallmarks of cancer: the next generation. Cell. 2011;144:646–674. 21376230 10.1016/j.cell.2011.02.013

[2] Speiser DE , Ho P-C, Verdeil G. Regulatory circuits of T cell function in cancer. Nat Rev Immunol. 2016;16:599–611. 27526640 10.1038/nri.2016.80

[3] Wang X et al Immunological therapy: a novel thriving area for triple-negative breast cancer treatment. Cancer Lett. 2019;442:409–428. 30419345 10.1016/j.canlet.2018.10.042

[4] Stagg J , Allard B. Immunotherapeutic approaches in triple-negative breast cancer: latest research and clinical prospects. Ther Adv Med Oncol. 2013;5:169–181. 23634195 10.1177/1758834012475152 PMC3630481

[5] Mullen G 3rd , et al (-)-Spiro[1-azabicyclo[2.2.2]octane-3,5′-oxazolidin-2′-one], a conformationally restricted analogue of acetylcholine, is a highly selective full agonist at the alpha 7 nicotinic acetylcholine receptor. J Med Chem. 2000;43:4045–4050. 11063601 10.1021/jm000249r

[6] Van Kampen M et al AR-R 17779 improves social recognition in rats by activation of nicotinic alpha7 receptors. Psychopharmacology (Berl). 2004;172:375–383. 14727003 10.1007/s00213-003-1668-7

[7] van Maanen MA et al Stimulation of nicotinic acetylcholine receptors attenuates collagen-induced arthritis in mice. Arthritis Rheum. 2009;60:114–122. 19116908 10.1002/art.24177

[8] Wang H et al Nicotinic acetylcholine receptor alpha7 subunit is an essential regulator of inflammation. Nature. 2003;421:384–388. 12508119 10.1038/nature01339

[9] de Jonge WJ et al Stimulation of the vagus nerve attenuates macrophage activation by activating the Jak2-STAT3 signaling pathway. Nat Immunol. 2005;6:844–851. 16025117 10.1038/ni1229

[10] Takahashi H , Ogata H, Nishigaki R, Broide DH, Karin M. Tobacco smoke promotes lung tumorigenesis by triggering IKKbeta- and JNK1-dependent inflammation. Cancer Cell. 2010;17:89–97. 20129250 10.1016/j.ccr.2009.12.008 PMC2818776

[11] Wang H et al Role of the nervous system in cancers: a review. Cell Death Discov. 2021;7:76. 33846291 10.1038/s41420-021-00450-y PMC8041826

[12] Orr-Urtreger A et al Mice deficient in the alpha7 neuronal nicotinic acetylcholine receptor lack alpha-bungarotoxin binding sites and hippocampal fast nicotinic currents. J Neurosci. 1997;17:9165–9171. 9364063 10.1523/JNEUROSCI.17-23-09165.1997 PMC6573618

[13] Ewens A , Mihich E, Ehrke MJ. Distant metastasis from subcutaneously grown E0771 medullary breast adenocarcinoma. Anticancer Res. 2005;25:3905–3915. 16312045

[14] Jensen MM , Jorgensen JT, Binderup T, Kjaer A. Tumor volume in subcutaneous mouse xenografts measured by microCT is more accurate and reproducible than determined by 18F-FDG-microPET or external caliper. BMC Med Imaging. 2008;8:16. 18925932 10.1186/1471-2342-8-16 PMC2575188

[15] Livak KJ , Schmittgen TD. Analysis of relative gene expression data using real-time quantitative PCR and the 2(-Delta Delta C(T)) Method. Methods. 2001;25:402–408. 11846609 10.1006/meth.2001.1262

[16] Amend SR , Valkenburg KC, Pienta KJ. Murine hind limb long bone dissection and bone marrow isolation. J Vis Exp. 2016;110:53936. 10.3791/53936 PMC4941920 27168390

[17] Helft J et al GM-CSF mouse bone marrow cultures comprise a heterogeneous population of CD11c(+)MHCII(+) macrophages and dendritic cells. Immunity. 2015;42:1197–1211. 26084029 10.1016/j.immuni.2015.05.018

[18] Hogquist KA et al T cell receptor antagonist peptides induce positive selection. Cell. 1994;76:17–27. 8287475 10.1016/0092-8674(94)90169-4

[19] Demaria S et al Immune-mediated inhibition of metastases after treatment with local radiation and CTLA-4 blockade in a mouse model of breast cancer. Clin Cancer Res. 2005;11:728–734. 15701862

[20] Chan T et al CHRFAM7A alters binding to the neuronal alpha-7 nicotinic acetylcholine receptor. Neurosci Lett. 2019;690:126–131. 30308236 10.1016/j.neulet.2018.10.010 PMC6320298

[21] Luboshits G et al Elevated expression of the CC chemokine regulated on activation, normal T cell expressed and secreted (RANTES) in advanced breast carcinoma. Cancer Res. 1999;59:4681–4687. 10493525

[22] Azenshtein E et al The CC chemokine RANTES in breast carcinoma progression: regulation of expression and potential mechanisms of promalignant activity. Cancer Res. 2002;62:1093–1102. 11861388

[23] Lyukmanova EN et al Human secreted proteins SLURP-1 and SLURP-2 control the growth of epithelial cancer cells via interactions with nicotinic acetylcholine receptors. Br J Pharmacol. 2018;175:1973–1986. 29505672 10.1111/bph.14194 PMC5980222

[24] Wu CH , Lee CH, Ho YS. Nicotinic acetylcholine receptor-based blockade: applications of molecular targets for cancer therapy. Clin Cancer Res. 2011;17:3533–3541. 21444681 10.1158/1078-0432.CCR-10-2434

[25] Ostrand-Rosenberg S , Sinha P, Beury DW, Clements VK. Cross-talk between myeloid-derived suppressor cells (MDSC), macrophages, and dendritic cells enhances tumor-induced immune suppression. Semin Cancer Biol. 2012;22:275–281. 22313874 10.1016/j.semcancer.2012.01.011 PMC3701942

[26] Shibata M et al Myeloid-derived suppressor cells: cancer, autoimmune diseases, and more. Oncotarget. 2022;13:1273–1285. 36395389 10.18632/oncotarget.28303 PMC9671473

[27] Bosiljcic M et al Targeting myeloid-derived suppressor cells in combination with primary mammary tumor resection reduces metastatic growth in the lungs. Breast Cancer Res. 2019;21:103. 31488209 10.1186/s13058-019-1189-x PMC6727565

[28] Peranzoni E et al Macrophages impede CD8 T cells from reaching tumor cells and limit the efficacy of anti-PD-1 treatment. Proc Natl Acad Sci U S A. 2018;115:E4041–E4050. 29632196 10.1073/pnas.1720948115 PMC5924916

[29] Arger NK et al CXCL9 and CXCL10 are differentially associated with systemic organ involvement and pulmonary disease severity in sarcoidosis. Respir Med. 2020;161:105822. 31783271 10.1016/j.rmed.2019.105822 PMC7028429

[30] Chen J et al IL-17 inhibits CXCL9/10-mediated recruitment of CD8(+) cytotoxic T cells and regulatory T cells to colorectal tumors. J Immunother Cancer. 2019;7:324. 31775909 10.1186/s40425-019-0757-z PMC6880503

[31] Hu H , Sun SC. Ubiquitin signaling in immune responses. Cell Res. 2016;26:457–483. 27012466 10.1038/cr.2016.40 PMC4822134

[32] Walseng E et al Ubiquitination regulates MHC class II-peptide complex retention and degradation in dendritic cells. Proc Natl Acad Sci U S A. 2010;107:20465–20470. 21059907 10.1073/pnas.1010990107 PMC2996684

[33] Jia L et al The roles of TNFAIP2 in cancers and infectious diseases. J Cell Mol Med. 2018;22:5188–5195. 30145807 10.1111/jcmm.13822 PMC6201362

[34] Pavlov VA , Chavan SS, Tracey KJ. Molecular and functional neuroscience in immunity. Annu Rev Immunol. 2018;36:783–812. 29677475 10.1146/annurev-immunol-042617-053158 PMC6057146

[35] Tracey KJ. Reflex control of immunity. Nat Rev Immunol. 2009;9:418–428. 19461672 10.1038/nri2566 PMC4535331

[36] Reardon C et al Lymphocyte-derived ACh regulates local innate but not adaptive immunity. Proc Natl Acad Sci U S A. 2013;110:1410–1415. 23297238 10.1073/pnas.1221655110 PMC3557089

[37] Rosas-Ballina M et al Acetylcholine-synthesizing T cells relay neural signals in a vagus nerve circuit. Science. 2011;334:98–101. 21921156 10.1126/science.1209985 PMC4548937

[38] Erin N , Duymus O, Ozturk S, Demir N. Activation of vagus nerve by semapimod alters substance P levels and decreases breast cancer metastasis. Regul Pept. 2012;179:101–108. 22982142 10.1016/j.regpep.2012.08.001

[39] Hato L et al Dendritic cells in cancer immunology and immunotherapy. Cancers. 2024;16:981. 38473341 10.3390/cancers16050981 PMC10930494

[40] Heras-Murillo I , Adán-Barrientos I, Galán M, Wculek SK, Sancho D. Dendritic cells as orchestrators of anticancer immunity and immunotherapy. Nat Rev Clin Oncol. 2024;21:257–277. 38326563 10.1038/s41571-024-00859-1

[41] Kawashima K , Fujii T, Moriwaki Y, Misawa H, Horiguchi K. Reconciling neuronally and nonneuronally derived acetylcholine in the regulation of immune function. Ann N Y Acad Sci. 2012;1261:7–17. 22823388 10.1111/j.1749-6632.2012.06516.x

[42] Olofsson PS et al alpha7 nicotinic acetylcholine receptor (alpha7nAChR) expression in bone marrow-derived non-T cells is required for the inflammatory reflex. Mol Med. 2012;18:539–543. 22183893 10.2119/molmed.2011.00405 PMC3356417

[43] Wilund KR et al Macrophages from alpha 7 nicotinic acetylcholine receptor knockout mice demonstrate increased cholesterol accumulation and decreased cellular paraoxonase expression: a possible link between the nervous system and atherosclerosis development. Biochem Biophys Res Commun. 2009;390:148–154. 19785985 10.1016/j.bbrc.2009.09.088

[44] van Maanen MA , Stoof SP, Larosa GJ, Vervoordeldonk MJ, Tak PP. Role of the cholinergic nervous system in rheumatoid arthritis: aggravation of arthritis in nicotinic acetylcholine receptor alpha7 subunit gene knockout mice. Ann Rheum Dis. 2010;69:1717–1723. 20511609 10.1136/ard.2009.118554

[45] The FO et al Activation of the cholinergic anti-inflammatory pathway ameliorates postoperative ileus in mice. Gastroenterology. 2007;133:1219–1228. 17919496 10.1053/j.gastro.2007.07.022

[46] Bruchfeld A et al Whole blood cytokine attenuation by cholinergic agonists ex vivo and relationship to vagus nerve activity in rheumatoid arthritis. J Intern Med. 2010;268:94–101. 20337855 10.1111/j.1365-2796.2010.02226.x PMC2937357

[47] van Maanen MA et al Two novel alpha7 nicotinic acetylcholine receptor ligands: in vitro properties and their efficacy in collagen-induced arthritis in mice. PLoS One. 2015;10:e0116227. 25617631 10.1371/journal.pone.0116227 PMC4305287

[48] Wang H et al Cholinergic agonists inhibit HMGB1 release and improve survival in experimental sepsis. Nat Med. 2004;10:1216–1221. 15502843 10.1038/nm1124

[49] Hao J et al Attenuation of CNS inflammatory responses by nicotine involves alpha7 and non-alpha7 nicotinic receptors. Exp Neurol. 2011;227:110–119. 20932827 10.1016/j.expneurol.2010.09.020 PMC3019302

[50] Simard AR et al Differential modulation of EAE by alpha9- and beta2-nicotinic acetylcholine receptors. Immunol Cell Biol. 2013;91:195–200. 23399696 10.1038/icb.2013.1 PMC3596513

[51] Liu J et al Syntenin1/MDA-9 (SDCBP) induces immune evasion in triple-negative breast cancer by upregulating PD-L1. Breast Cancer Res Treat. 2018;171:345–357. 29845474 10.1007/s10549-018-4833-8

[52] Sun W et al Synergistic triple-combination therapy with hyaluronic acid-shelled PPy/CPT nanoparticles results in tumor regression and prevents tumor recurrence and metastasis in 4T1 breast cancer. Biomaterials. 2019;217:119264. 31260883 10.1016/j.biomaterials.2019.119264

[53] Duan X et al Photodynamic therapy mediated by nontoxic core-shell nanoparticles synergizes with immune checkpoint blockade to elicit antitumor immunity and antimetastatic effect on breast cancer. J Am Chem Soc. 2016;138:16686–16695. 27976881 10.1021/jacs.6b09538 PMC5667903

